# Factors associated with fatigue in CNS inflammatory diseases with AQP4 and MOG antibodies

**DOI:** 10.1002/acn3.51008

**Published:** 2020-03-18

**Authors:** Tianrong Yeo, Giordani Rodrigues dos Passos, Louwai Muhammed, Rosie Everett, Sandra Reeve, Silvia Messina, Fay Probert, Maria Isabel Leite, Jacqueline Palace

**Affiliations:** ^1^ Department of Pharmacology University of Oxford Oxford UK; ^2^ Department of Neurology National Neuroscience Institute Singapore Singapore; ^3^ Nuffield Department of Clinical Neurosciences John Radcliffe Hospital University of Oxford Oxford UK; ^4^ Department of Neurology Sao Lucas Hospital Pontifical Catholic University of Rio Grande do Sul Porto Alegre Brazil; ^5^ Department of Neurology Charing Cross Hospital London UK; ^6^ Department of Clinical Neurology John Radcliffe Hospital Oxford University Hospitals Trust Oxford UK

## Abstract

**Objective:**

Fatigue is a common and disabling symptom amongst people with multiple sclerosis, however it has not been compared across the central nervous system (CNS) inflammatory diseases associated with aquaporin‐4 (AQP4) and myelin oligodendrocyte glycoprotein (MOG) antibodies (Ab). We explored the factors associated with fatigue within and across the two diseases, and compared fatigue levels between them.

**Methods:**

We performed a cross‐sectional study of 90 AQP4‐Ab and 44 MOG‐Ab patients. Fatigue was assessed using the Modified Fatigue Impact Scale (MFIS). Clinical, demographic, and psychometric (anxiety, depression, pain) data were used as independent variables. Multivariable linear regression was used to identify significant independent variables associated with fatigue within and across the two diseases.

**Results:**

Within AQP4‐Ab patients, age (*P* = 0.002), disease duration (*P* = 0.004), number of clinical attacks (*P* = 0.001), disability (*P* = 0.007), pain interference (*P* < 0.001), anxiety (*P* = 0.026), and depression (*P* < 0.001) were significant independent variables. Interestingly, disease duration had a negative association with fatigue (*P* = 0.004). Within MOG‐Ab patients, pain interference score (*P* < 0.001) and anxiety (*P* = 0.001) were significant independent variables. Although fatigue was worse in AQP4‐Ab patients compared to MOG‐Ab patients (*P* = 0.008) in all patients as well as in those who ever had transverse myelitis (*P* = 0.023), this was driven by the differences in age, disability and pain interference rather than antibody subtype itself.

**Interpretation:**

Multiple factors, but not the antibody specificity, appear to contribute to fatigue in antibody positive CNS inflammatory diseases. A multifaceted treatment approach is needed to better manage the physical, cognitive, and psychosocial aspects of fatigue in these patients.

## Introduction

Fatigue is a debilitating yet common symptom affecting about 66% to 75% of individuals with multiple sclerosis (MS).^1^, ^2^, ^3^, ^4^ It can present early on in the disease course;^5^ indeed the presence of fatigue in those with clinically isolated syndromes is an independent risk factor for the development of clinically definite MS,^6^ although the mechanisms by which fatigue arises are not completely understood.^7^, ^8^

In the neuromyelitis optica spectrum disorders (NMOSD) clinic, fatigue is also a frequent complaint. This observation is supported by a few studies to date that seem to suggest that fatigue in NMOSD is as prevalent and as severe as that seen in MS.^9^, ^10^, ^11^, ^12^ There are a number of potential pathogenic factors that could contribute to fatigue in NMOSD such as astrocytic injury, demyelination, and axonal loss, as well as secondary contributors like disability, pain, depression, and side effects of medications. Some studies have identified a correlation between fatigue and depression in NMOSD,^9^, ^10^, ^13^ however the small number of patients (range 33–40) within these studies mean that adjustment for covariates to explore independent contributors of fatigue was not possible.^9^, ^10^, ^12^, ^13^, ^14^ Furthermore, the analyses within these studies were performed by combining not only patients who were aquaporin‐4‐antibody (AQP4‐Ab) positive but also ‘seronegative’ NMOSD patients,^9^, ^11^, ^12^, ^13^, ^14^, ^15^ which is likely to comprise different pathological conditions including myelin oligodendrocyte glycoprotein‐antibody (MOG‐Ab) disease, atypical MS and other autoimmune and connective tissue diseases. We have previously noted that there are different drivers of pain in AQP4‐Ab disease compared to antibody negative NMOSD,^16^ thus is important to separate different diseases when trying to understand the drivers of symptoms. Furthermore, the study of specific diseases allows us to explore whether the differing central nervous system (CNS) targets that is, astrocytes or myelin/oligodendrocytes can affect the mechanism/s of fatigue. Identification of the common and differing contributors of fatigue within each specific CNS inflammatory disease would hence allow for a more focused management of this symptom.

In this cross‐sectional study, using the Modified Fatigue Impact Scale (MFIS), we explored the factors associated with fatigue within and across those with antibodies to AQP4 (which is astrocyte situated) and those with antibodies to MOG (a myelin‐based antigen expressed by oligodendrocytes), and compared fatigue levels between them.

## Methods

### Patients

All patients were recruited from the Oxford national NMO service at the John Radcliffe Hospital. Consented patients over the age of 16 with AQP4‐Ab or MOG‐Ab (tested by cell‐based assays as previously described),^17^ and at least 1 clinical event consistent with CNS inflammation were shortlisted.

Clinical and demographic data was obtained from medical notes, referenced to the time point at which MFIS was assessed. These included age, gender, ethnicity, disease duration (disease onset to time of MFIS assessment), attack phenotype (i.e., monofocal vs. multifocal involvement, on an “ever had” basis), number of attacks (cumulative), disability as measured by the European Database for Multiple Sclerosis (EDMUS) scale at the time of MFIS assessment, presence of severe attacks (i.e., EDMUS of ≥6, and/or visual acuity of ≤0.1 at nadir of any attack), current body mass index (BMI), current usage of fatigue‐inducing medications (Supplemental Table S1), and current presence of fatigue‐inducing comorbidities (Supplemental Table S1). The EDMUS scale is derived from the Expanded Disability Status Scale (EDSS),^18^ with a similar range from 0 to 10 but only including integers.^19^ It describes the function of the patient and hence it is easier to score without a full neurological examination but has been shown to have excellent correlation with the equivalent score in the EDSS*.* ^19^

All patients consented for the NMO tissue bank which was approved by the Oxford Research Ethics Committee C (Ref: 10/H0606/56 and 16/SC/0224A).

### Instruments

This was a cross‐sectional study carried out during the patients’ clinical visit. Fatigue impact was assessed by the MFIS, a widely used self‐report instrument in MS fatigue research,^8^, ^20^, ^21^ with good validity and reliability demonstrated in both MS and NMOSD.^14^, ^22^, ^23^ The MFIS also shows strong correlation with another commonly used instrument, the Fatigue Severity Scale,^24^, ^25^, ^26^ indicating that they are measuring similar constructs.^22^, ^26^, ^27^ The MFIS consists of a total of 21 items each scored on a Likert scale (0–4), grouped into three subscales; physical (9 items), cognitive (10 items), and psychosocial (2 items), adding up to a maximum fatigue score of 84. Pain was measured by the Brief Pain Inventory (BPI), which consists of items scored on a Likert scale (0–10), aggregated into two components; pain inference and pain severity. The final scores are the average scores of the items within each component, with a score range of 0 to 10 (10 being worst). Anxiety and depression were determined using the Hospital Anxiety and Depression Scale (HADS).^28^ Each construct is measured by 7 items on a Likert scale (0–3), with the total score being the sum of individual item scores (score range of 0 to 21 within each construct). Higher scores indicate increased anxiety and depression.

As pain, anxiety, and depression are postulated to contribute to fatigue, all patients in this study had instruments measuring these constructs administered at the same time point (i.e., there was no missing data for all instruments for all patients).

### Statistical analysis

All analyses were performed with STATA software (Release 14, College Station, TX: Statacorp LP). The MFIS score (dependent outcome variable) was analysed as a continuous variable and its internal consistency was determined by Cronbach’s alpha. Other instrument scores were also analysed as continuous variables. Comparative analyses between AQP4‐Ab and MOG‐Ab patients were performed using Mann‐Whitney *U* test or two‐sample *t*‐test as appropriate for continuous variables, and with Chi squared test for categorical variables. Two‐tailed *P* values of <0.05 were considered statistically significant. Univariable linear regression was first used to explore each independent variable in ‘predicting’ fatigue for each of the two disease groups, using the MFIS total score as the dependent variable. To create a clinically relevant yet parsimonious model with a low risk of multicollinearity, all clinical, demographic, and instrument data mentioned in the above sections were included as independent variables in a multivariable linear regression model. This is followed by a backward stepwise elimination strategy whereby the least significant independent variable was removed at each step. The final model consisted only of independent variables with *P* < 0.05. This was done for each antibody disease separately, and then repeated again for the whole cohort using only those independent variables significant within the multivariable model of each disease, with the addition of the antibody subtype.

## Results

### Demographic and clinical characteristics

A total of 134 patients were included; 90 AQP4‐Ab and 44 MOG‐Ab patients. Compared to MOG‐Ab patients, as expected, AQP4‐Ab patients were older (mean [SD], 53.7 [16.7] years vs. 38.9 [14.4] years; *P* < 0.001), consisted of a higher proportion of females (74/90 vs. 27/44; *P* = 0.008) and non‐whites (40/90 vs. 5/44; *P* < 0.001), had longer disease duration (median [range], 6.13 [0.01–38.1] years vs. 2.14 [0.06–37.4] years; *P* = 0.002), and were more disabled as measured by the EDMUS scale (median [range], 3 [0–8] vs. 1 [0–6]; *P* < 0.001) (Table 1). More AQP4‐Ab patients were also on fatigue‐inducing medications (50/90 vs. 5/44; *P* < 0.001) and a greater proportion had fatigue‐inducing comorbidities (27/90 vs. 3/44; *P* = 0.003) (Table 1).

**Table 1:** Demographic and clinical data of patients grouped by antibody diagnosis.

	AQP4‐Ab (*n* = 90)	MOG‐Ab (*n* = 44)	*P* value
Age at MFIS assessment, mean (SD), years	53.7 (16.7)	38.9 (14.4)	<0.001
Female, No. (%)	74 (82.2)	27 (61.4)	0.008
Ethnicity, No. (%)			<0.001
White	50 (55.6)	39 (88.6)	
Non‐white	40 (44.4)	5 (11.4)	
Disease duration, median (range), years	6.13 (0.01 to 38.1)	2.14 (0.06 to 37.4)	0.002
Attack phenotype, No. (%)			0.891
Monofocal	40 (44.4)	19 (43.2)	
Multifocal	50 (55.6)	25 (56.8)	
Number of clinical attack/s, median (range)	2 (1 to 19)	2 (1 to 11)	0.146
Presence of severe attack/s, No. (%)^1^	71 (78.9)	31 (77.5)	0.859
EDMUS scale, median (range)	3 (0 to 8)	1 (0 to 6)	<0.001
Current BMI, median (range)	26.9 (17.8 to 50.8)	27.6 (19.5 to 43.7)	0.775
Fatigue‐inducing medications, No. (%)	50 (55.6)	5 (11.4)	<0.001
Fatigue‐inducing comorbidities, No. (%)	27 (30.0)	3 (6.8)	0.003

Ab, antibody; AQP4, aquaporin‐4; BMI, body mass index; EDMUS, European Database for Multiple Sclerosis; MFIS, Modified Fatigue Impact Scale; MOG, myelin oligodendrocyte glycoprotein.

^1^Defined as EDMUS of ≥6, and/or visual acuity of ≤0.1 at nadir of any attack.

### Instrument scores

The MFIS score showed excellent internal consistency within the entire cohort of patients, as shown by high Cronbach’s alpha of 0.97 for MFIS total score, 0.96 for physical subscale, 0.96 for cognitive subscale, and 0.83 for psychosocial subscale.

MFIS scores (total, physical and psychosocial components) were significantly higher in AQP4‐Ab compared to MOG‐Ab patients, as were pain interference and pain severity scores, while no significant differences were observed in anxiety (HADS‐A) and depression (HADS‐D) scores (Table 2).

**Table 2:** Instrument data of patients grouped by antibody diagnosis.

	AQP4‐Ab (*n* = 90)	MOG‐Ab (*n* = 44)	*P* value
MFIS score, mean (SD)			
Total (/84)	37.3 (21.8)	26.8 (20.0)	0.008
Physical (/36)	18.8 (10.8)	13.6 (9.9)	0.008
Cognitive (/40)	14.8 (10.3)	11.4 (9.3)	0.063
Psychosocial (/8)	3.7 (2.4)	2.6 (2.5)	0.015
MFIS total score ≥38, No. (%)^1^	52 (57.8)	13 (29.5)	0.002
Pain severity score (/10), median (range)	3.5 (0.0 to 10.0)	0.5 (0.0 to 6.5)	<0.001
Pain interference score (/10), median (range)	3.1 (0.0 to 9.6)	0.0 (0.0 to 9.7)	0.002
HADS‐A (/21), median (range)	6.0 (0.0 to 21)	6.0 (0.0 to 15)	0.992
HADS‐D (/21), median (range)	5.0 (0.0 to 17)	3.0 (0.0 to 15)	0.233

Ab, antibody; AQP4, aquaporin‐4; HADS‐A, Hospital Anxiety and Depression Scale‐Anxiety; HADS‐D, Hospital Anxiety and Depression Scale‐Depression; MFIS, Modified Fatigue Impact Scale; MOG, myelin oligodendrocyte glycoprotein.

^1^Cut‐off of 38 was proposed in a study by Flachenecker, et al.^27^

### Factors associated with fatigue in AQP4‐Ab patients

On univariable linear regression analyses, older age, white ethnicity, presence of severe attacks, higher EDMUS scale, higher BMI, usage of fatigue‐inducing medications, higher pain severity and pain interference scores, and higher HADS‐A and HADS‐D were significant factors associated with fatigue in AQP4‐Ab patients (Table 3).

**Table 3:** Univariable linear regression analysis (MFIS total score) within AQP4‐Ab patients.

Independent variable	Regression coefficient, *B*	95% CI	*P* value	R^2^
Age at MFIS assessment	0.364	0.099 to 0.630	0.008	0.078
Gender (Female)	−2.206	−14.227 to 9.815	0.716	0.002
Ethnicity (Non‐White)	−9.785	−18.806 to −0.764	0.034	0.050
Disease duration	−0.070	−0.706 to 0.567	0.828	0.001
Attack phenotype (Multifocal)	4.520	−4.686 to 13.726	0.332	0.011
Number of clinical attack/s	0.808	−0.660 to 2.277	0.277	0.013
Presence of severe attack/s^1^	12.070	1.093 to 23.046	0.032	0.052
EDMUS scale	5.430	3.659 to 7.200	<0.001	0.297
Current BMI	0.789	0.102 to 1.477	0.025	0.065
Fatigue‐inducing medications	16.715	8.163 to 25.267	<0.001	0.146
Fatigue‐inducing comorbidities	4.741	−5.246 to 14.727	0.348	0.010
Pain severity score	3.829	2.528 to 5.129	<0.001	0.280
Pain interference score	4.925	3.813 to 6.037	<0.001	0.468
HADS‐A	2.904	2.129 to 3.679	<0.001	0.387
HADS‐D	3.551	2.859 to 4.243	<0.001	0.542

Ab, antibody; AQP4, aquaporin‐4; BMI, body mass index; EDMUS, European Database for Multiple Sclerosis; HADS‐A, Hospital Anxiety and Depression Scale‐Anxiety; HADS‐D, Hospital Anxiety and Depression Scale‐Depression; MFIS, Modified Fatigue Impact Scale.

^1^Defined as EDMUS of ≥6, and/or visual acuity of ≤0.1 at nadir of any attack.

In multivariable linear regression analysis using step‐wise backward elimination, older age, shorter disease duration, higher number of clinical attacks, higher EDMUS scale, higher pain interference score, higher HADS‐A, and higher HADS‐D were identified as significant independent variables (Table 5). All independent variables in this model had *P* values <0.05. The adjusted R^2^ for this final model was 0.77. In view of the negative regression coefficient of disease duration in the final model, a multicollinearity check performed revealed that the variance inflation factor (VIF) scores of all significant predictors were <3, with a mean of 2.05, denoting a low risk of multicollinearity.^29^

### Factors associated with fatigue in MOG‐Ab patients

On univariable linear regression analyses, higher BMI, usage of fatigue‐inducing medications, presence of fatigue‐inducing comorbidities, higher pain severity score and pain interference score, higher HADS‐A and HADS‐D scores were significant factors associated with fatigue in MOG‐Ab patients (Table 4).

**Table 4:** Univariable linear regression analysis (MFIS total score) within MOG‐Ab patients.

Independent variable	Regression coefficient, *B*	95% CI	*P* value	R^2^
Age at MFIS assessment	0.005	−0.426 to 0.437	0.980	<0.001
Gender (Female)	0.529	−12.096 to 13.155	0.933	<0.001
Ethnicity (Non‐White)	3.615	−15.724 to 22.954	0.708	0.003
Disease duration	0.333	−0.402 to 1.068	0.366	0.020
Attack phenotype (Multifocal)	1.307	−11.098 to 13.713	0.833	0.001
Number of clinical attack/s	1.044	−1.802 to 3.891	0.463	0.013
Presence of severe attack/s^1^	−4.097	−19.719 to 11.526	0.599	0.007
EDMUS scale	2.776	−1.733 to 7.286	0.221	0.036
Current BMI	1.129	0.027 to 2.231	0.045	0.104
Fatigue‐inducing medications	19.636	1.254 to 38.017	0.037	0.010
Fatigue‐inducing comorbidities	24.902	1.777 to 48.028	0.035	0.101
Pain severity score	5.231	2.430 to 8.031	0.001	0.253
Pain interference score	5.526	3.782 to 7.269	<0.001	0.494
HADS‐A	2.257	0.774 to 3.741	0.004	0.183
HADS‐D	2.963	1.861 to 4.066	<0.001	0.412

Ab, antibody; BMI, body mass index; EDMUS, European Database for Multiple Sclerosis; HADS‐A, Hospital Anxiety and Depression Scale‐Anxiety; HADS‐D, Hospital Anxiety and Depression Scale‐Depression; MFIS, Modified Fatigue Impact Scale; MOG, myelin oligodendrocyte glycoprotein

^1^Defined as EDMUS of ≥6, and/or visual acuity of ≤0.1 at nadir of any attack.

Using the same multivariable linear regression strategy as specified above, higher pain interference score and higher HADS‐A were identified as significant independent variables (Table 5). Both variables in this model had *P* values <0.05. The adjusted R^2^ for this final model was 0.59. The VIF scores of both significant predictors were 1.02, indicating a very low risk of multicollinearity.^29^

**Table 5:** Multivariable linear regression models (MFIS total score) within AQP4‐Ab and MOG‐Ab patients separately, and as a combined cohort.

	Independent variable	Regression coefficient, *B*	95% CI	*P* value
AQP4‐Ab	Age at MFIS assessment	0.299	0.114 to 0.483	0.002
	Disease duration	−0.616	−0.197 to −1.035	0.004
	Number of clinical attack/s	1.876	0.795 to 2.956	0.001
	EDMUS scale	1.907	0.534 to 3.281	0.007
	Pain interference score	2.430	1.414 to 3.447	<0.001
	HADS‐A	0.779	0.094 to 1.465	0.026
	HADS‐D	1.641	0.925 to 2.358	<0.001
MOG‐Ab	Pain interference score	5.166	3.597 to 6.736	<0.001
	HADS‐A	1.792	0.740 to 2.844	0.001
Whole cohort	Age at MFIS assessment	0.211	0.056 to 0.366	0.008
	Disease duration	−0.501	−0.839 to −0.163	0.004
	Number of clinical attack/s	1.513	0.543 to 2.484	0.003
	EDMUS scale	1.766	0.504 to 3.029	0.006
	Pain interference score	2.841	1.905 to 3.776	<0.001
	HADS‐A	0.813	0.205 to 1.421	0.009
	HADS‐D	1.552	0.887 to 2.216	<0.001
	Antibody diagnosis	2.294	−2.684 to 7.272	0.363

Antibody diagnosis: AQP4‐Ab = 0, MOG‐Ab = 1.

Ab, antibody; EDMUS, European Database for Multiple Sclerosis; HADS‐A, Hospital Anxiety and Depression Scale‐Anxiety; HADS‐D, Hospital Anxiety and Depression Scale‐Depression; MFIS, Modified Fatigue Impact Scale; MOG, myelin oligodendrocyte glycoprotein.

### Factors associated with fatigue across all antibody positive patients

As shown in Table 2, the MFIS total score was higher in all AQP4‐Ab patients compared to all MOG‐Ab patients. We observed that this was also the case within patients who ever had transverse myelitis (TM); AQP4‐Ab TM patients had higher MFIS total scores compared to MOG‐Ab TM patients (mean [SD], 38.2 [21.1] vs. 26.9 [21.8]; *P* = 0.023). However, the factors associated with fatigue differed between the two disease groups, thus in order to identify if the antibody specificity itself influenced fatigue, we performed multivariable linear regression on all the patients by including the significant factors identified from the within disease multivariable linear regression models (Table 5), with the addition of antibody diagnosis, as independent variables. Older age, shorter disease duration, higher number of clinical attacks, higher EDMUS scale, higher pain interference score, higher HADS‐A and higher HADS‐D remained as significant independent variables (all *P* < 0.05), whereas antibody diagnosis was not (*P* = 0.363) (Table 5). To investigate if antibody diagnosis was a significant factor associated with fatigue in patients without optic neuritis alone phenotypes (optic neuritis alone phenotype being more common in MOG‐Ab disease, that is, 36.4% vs. 13.3% in AQP4‐Ab disease, and may be less likely to cause fatigue), we restricted this analysis to those who ever had TM. The same factors remained significant (*P* < 0.05) with the exception of EDMUS scale (*P* = 0.052), while antibody diagnosis was again not a significant independent variable (*P* = 0.707).

We further extended the above multivariable model (combined cohort, as shown in Table 5) by including the multiplicative interactions between antibody diagnosis and the other independent variables (Supplemental Table S2). None of the multiplicative interactions was significant, except for pain interference score with antibody diagnosis (*P*_interaction_ = 0.034). This result implies that if all other variables in the model were kept constant, MOG‐Ab patients have an increase of 2.325 points more on the MFIS total score for every 1‐point increase in the pain interference score, as compared to AQP4‐Ab patients. In other words, the effect of pain interference on fatigue is more pronounced in MOG‐Ab patients. Of note, all the significant independent variables from the regression model without interaction analyses were still significant in this model, while antibody diagnosis itself as an independent variable remained nonsignificant. We also ran a multivariable linear regression analysis of the combined cohort using all the variables available and notably, the significant variables identified in Table 5 (using within disease significant variables) retained significance.

### Analysis of MFIS subscales

Because in theory, different factors could drive the different components of the MFIS (e.g., the physical subscale may be driven more by EDMUS scale, while the cognitive subscale by pain interference score or HADS), we also analyzed these two subscales separately. Supplemental Tables S3 and S4 show that there are no major differences in pattern except for anxiety being associated with the cognitive subscale but not with the physical component for the whole cohort.

## Discussion

In this cross‐sectional study, we identified clinically relevant factors associated with fatigue within and across AQP4‐Ab and MOG‐Ab CNS inflammatory diseases, and compared the fatigue levels between the two diseases. We found that age, number of clinical attacks, disability, pain interference, anxiety, and depression were associated with fatigue (in a positive direction) in AQP4‐Ab patients. Interestingly, disease duration had a negative association with fatigue, that is, increased disease duration was associated with lower MFIS scores. Within MOG‐Ab patients, pain interference and anxiety were associated with fatigue, in a positive direction. Although fatigue was worse in AQP4‐Ab patients compared to MOG‐Ab patients in all patients as well as in those who had TM, it appeared that the differences in age, disability, and pain interference (i.e., these are factors associated with fatigue as well as baseline differentiators between AQP4‐Ab and MOG‐Ab patients) rather than antibody subtype itself was driving this difference as antibody diagnosis was not a significant independent variable once these factors were accounted for.

Previous studies on NMOSD patients have demonstrated a correlation between fatigue and depression.^9^, ^10^, ^13^ Our findings on univariable analysis in both AQP4‐Ab and MOG‐Ab patients are in agreement with this observation, although on multivariable analysis, depression remained a significant independent variable only within AQP4‐Ab patients. Other studies in NMOSD cohorts that consisted predominantly of AQP4‐Ab patients have reported that disability (measured by the EDSS) and disease duration were not correlated with fatigue.^10^, ^13^ This is in contrast to our findings as we found that disability and disease duration had significant positive and negative associations with fatigue respectively. These differences could be due to the smaller sample size with inclusion of patients without AQP4‐Ab, analyzing fatigue as a binary variable (using a cut‐off score) in those studies, as well as the lack of adjustment for covariates – an important consideration given that covariates may alter the effect of the exploratory predictor in fatigue research. This is highlighted by one study that supported our findings in which EDSS was associated with fatigue, even after correction for age and disease duration,^11^ although the study also included AQP4‐Ab negative patients. We also observed that within MOG‐Ab patients, disability was not a contributing factor to fatigue as most MOG‐Ab patients had recovered to low disability states at the time of MFIS assessment. We also found that pain severity was not a contributory factor in both AQ4‐Ab and MOG‐Ab patients, consistent with a previous study showing that pain severity was not significantly higher in fatigued versus nonfatigued AQP4‐Ab patients.^10^

An interesting observation in our study was that disease duration had a negative association with fatigue within AQP4‐Ab patients, even though the regression coefficient is not exceedingly large. This could be explained by an adaptive process within the patient to better manage the physical, cognitive, and psychosocial aspects of fatigue with time. This is highly encouraging as it suggests that improvement of fatigue can occur and perhaps even accelerated by pharmacotherapy (although none of our patients was on medications for fatigue, for example, modafinil or amantadine, to explore this) as well as nonpharmacological measures, targeted at the contributors of fatigue (e.g., pain, anxiety, depression). Assuming that primary CNS fatigue is related to active CNS inflammation, our observation of a reduction in fatigue over time supports the fact that chronic inflammation and progression outside of a relapse is typical in MS and atypical in NMOSD.^30^ We also found the antibody diagnosis was not a determinant of fatigue within all antibody positive patients. This infers that the CNS target (i.e., astrocyte or myelin based) is not a driver of fatigue when other factors (age, disease duration, number of clinical attacks, disability, pain interference, anxiety, and depression) were considered.

Within AQP4‐Ab patients, while the relationship of pain, disability, age, disease duration, and number of clinical attacks with fatigue is clear, this is less so for anxiety and depression. Although anxiety and depression can certainly contribute to fatigue, a reverse association cannot be excluded, as they can be a consequence of the impact of fatigue on patients. Indeed, the relationship between these factors (fatigue impact, anxiety, depression, and pain interference) could exist in a bidirectional feedback loop. Regardless, this informs that the treatment of fatigue must be holistic and multifaceted, involving psychotherapy (e.g., cognitive behavioral therapy) and pharmacotherapy,^7^, ^31^ aimed at reducing anxiety, depression, and pain interference. This approach can be extended to MOG‐Ab patients, as pain interference and anxiety are the only significant factors associated with fatigue. Our findings also highlight the importance of preventing relapses and residual disability as increased number of attacks and higher EDMUS scale predict fatigue within AQP4‐Ab patients.

Our study has several notable strengths. This is the largest study of fatigue in AQP4‐Ab patients and also represents the first attempt to quantify and investigate fatigue within MOG‐Ab patients. Furthermore, we have no missing data for all instruments, which were collected at the same time point, and explored a comprehensive list of clinically relevant variables as independent variables. We do however acknowledge a few limitations of our study. We did not include age and gender‐matched healthy or other fatigue‐related disease controls such as MS. It has however been shown that fatigue levels are higher in NMOSD patients compared to healthy controls.^13^, ^14^ Indeed, previous MS studies conducted in Western Europe have reported a median MFIS (total) score ranging from 11 to 20,^23^, ^26^ and a mean score of 16.5 within healthy controls,^8^ with the mean age ranging from 36.1 to 50.6 years (comparable to our patient cohort) in those studies. This suggests that the MFIS total scores we observed in MOG‐Ab (mean 26.8) and AQP4‐Ab (mean 37.3) are higher than that of healthy individuals. Also, we did not collect an extensive list of conventional MRI parameters to avoid over‐fitting in our multivariable models. Moreover, it has been reported that there were no differences in brain (number of lesions) and spinal cord (number of segments containing lesions) abnormalities in comparing NMOSD patients with fatigue to those without fatigue.^13^, ^14^

In conclusion, we have identified that multiple factors contribute to fatigue in AQP4‐Ab and MOG‐Ab disease, and that fatigue is more severe in AQP4‐Ab compared to MOG‐Ab largely driven by the differences in age, disability, and pain interference between the two diseases. There is a need for the neurologist to be aware and adopt a proactive treatment approach to manage the physical, cognitive, and psychosocial aspects of fatigue. Future work will investigate fatigue longitudinally, adjusted for covariates in a time‐dependent manner, to explore how fatigue levels change with time and with treatment. This will also help to address the temporal association of fatigue with depression and anxiety. Advanced MRI techniques can also be employed to better delineate the neural basis that underlie fatigue within antibody positive CNS inflammatory diseases, and determine the correlation of MRI measures with fatigue severity for biomarker discovery. Additionally, contrasting and comparing with other diseases that cause fatigue, particularly MS, may identify generic and disease‐specific contributors of fatigue to help understand the etiology of fatigue better.

## Author Contributions

T.Y. and J.P. contributed to the conception and design of the study. T.Y., G.R.P., L.M., R.E., S.R., S.M., F.P., M.I.L., and J.P. contributed to the acquisition and analysis of data. T.Y. and J.P. contributed to drafting the text and preparing the tables.

## Conflicts of Interest

L.M., R.E., S.R., and F.P. report no disclosures relevant to the manuscript. T.Y. has received travel grants from UCB, Merck and PACTRIMS, and travel awards from ACTRIMS and Orebro University. G.R.P. has received scholarships from ECTRIMS, World Federation of Neurology and Novartis; funding for research from Biogen, Novartis, and Roche; travel grants from Roche, Sanofi‐Genzyme, and Teva; and fees for editorial content from Bayer, Merck Serono, and Roche. S.M. has received travel grants from Biogen, Novartis, Bayer, Merck, Almirall and honorarium for advisory work from Biogen. M.I.L. reports being involved in aquaporin‐4 testing, receiving support from the National Health Service National Specialised Commissioning Group for Neuromyelitis Optica and the National Institute for Health Research Oxford Biomedical Research Centre, receiving speaking honoraria from Biogen Idec, and receiving travel grants from Novartis. J.P. is partly funded by highly specialized services to run a national congenital myasthenia service and a neuromyelitis service. She has received support for scientific meetings and honorariums for advisory work from Merck Serono, Biogen Idec, Novartis, Teva, Chugai Pharma and Bayer Schering, Alexion, Roche, Genzyme, MedImmune, EuroImmun, MedDay, Abide and ARGENX, and grants from Merck Serono, Novartis, Biogen Idec, Teva, Abide, and Bayer Schering. Her hospital trust received funds for her role as the clinical lead for the RSS, and she has received grants from the MS society, Guthy Jackson Foundation, NIHR, Oxford Health Services Research Committee, EDEN, MRC, and John Fell for research studies.

## Supporting information


**Table S1**. List of fatigue‐inducing medications used and fatigue‐inducing comorbidities present in the patient cohort.
**Table S2**. Multivariable linear regression model (MFIS total score) of the combined cohort, with multiplicative interaction between antibody diagnosis and other independent variables.
**Table S3**. Multivariable linear regression analysis (MFIS physical subscale) within AQP4‐Ab and MOG‐Ab patients separately, and as a combined cohort.
**Table S4**. Multivariable linear regression analysis (MFIS cognitive subscale) within AQP4‐Ab and MOG‐Ab patients separately, and as a combined cohort.
