# Supplementary material for: Factors associated with fatigue in CNS inflammatory diseases with AQP4 and MOG antibodies
Source: Ann Clin Transl Neurol. 2020 Mar 18;7(3):375–83. doi: 10.1002/acn3.51008 (PMC7086003; doi:10.1002/acn3.51008)
Supplement: Supplementary file 1 — Table S1. List of fatigue‐inducing medications used and fatigue‐inducing comorbidities present in the patient cohort. Table S2. Multivariable linear regression model (MFIS total score) of the combined cohort, with multiplicative interaction between antibody diagnosis and other independent variables. Table S3. Multivariable linear regression analysis (MFIS physical subscale) within AQP4‐Ab and MOG‐Ab patients separately, and as a combined cohort. Table S4. Multivariable linear regression analysis (MFIS cognitive subscale) within AQP4‐Ab and MOG‐Ab patients separately, and as a combined cohort. [file ACN3-7-375-s001.docx]

**Table S1. List of fatigue-inducing medications used and fatigue-inducing comorbidities present in the patient cohort**

| Medications | Anti-depressants | - Amitriptyline |
| --- | --- | --- |
|  |  | - Venlafaxine |
|  |  | - Fluoxetine |
|  |  | - Sertraline |
|  | Anti-epileptics | - Carbamazepine |
|  |  | - Oxcarbazepine |
|  |  | - Lamotrigine |
|  |  | - Topiramate |
|  | GABAergics | - Gabapentin |
|  |  | - Pregabalin |
|  | Benzodiazepines/hypnotics | - Diazepam |
|  |  | - Zopiclone |
|  | Anti-cholinergics | - Solifenacin |
|  |  | - Oxybutynin |
|  |  | - Tolterodine |
|  | Anti-spasticity | - Baclofen |
|  |  | - Tizanidine |
|  | Opiates/opioids | - Morphine |
|  |  | - Codeine |
|  |  | - Tramadol |
|  |  |  |
| Comorbidities | Concomitant autoimmune diseases | - Rheumatoid arthritis |
|  |  | - Connective tissue diseases (Systemic lupus erythematosus, Sjogren’s syndrome) |
|  |  | - Autoimmune hypothyroidism |
|  |  | - Myaesthenia gravis |
|  |  | - Primary sclerosing cholangitis |
|  | Metabolic disorders | - Diabetes mellitus - Current anaemia, defined as haemoglobin less than 110 g/L |
|  | Active cancer, involving any organ | - Oesophageal cancer - Basal cell carcinoma - Lymphoma |
|  | Sleep disorders | - Obstructive sleep apnoea |
|  | Heart disease | - Ischaemic heart disease |
|  | Chronic lung disease (excluding asthma), from any cause | - Bronchiectasis - Pulmonary fibrosis |
|  | Chronic liver disease, from any cause | - Non-alcoholic steatohepatitis |
|  | Renal failure, from any cause | - Nephrectomy from previous renal cancer |
|  | Other CNS disorders | - Ischaemic stroke - Subarachnoid haemorrhage |

GABA = gamma-aminobutyric acid oligodendrocyte glycoprotein.

**Table S2. Multivariable linear regression model (MFIS total score) of the combined cohort, with multiplicative interaction between antibody diagnosis and other independent variables**

|  | Independent variable | Regression coefficient, *B* | 95% CI | p value |
| --- | --- | --- | --- | --- |
| Whole cohort | Age at MFIS assessment | 0.299 | 0.099 to 0.498 | 0.004 |
|  | Age at MFIS assessment * Antibody diagnosis | -0.274 | -0.596 to 0.047 | 0.094 |
|  | Disease duration | -0.616 | -1.071 to -0.161 | 0.008 |
|  | Disease duration * Antibody diagnosis | 0.235 | -0.445 to 0.915 | 0.495 |
|  | Number of clinical attack/s | 1.876 | 0.703 to 3.048 | 0.002 |
|  | Number of clinical attack/s * Antibody diagnosis | -0.841 | -3.174 to 1.492 | 0.477 |
|  | EDMUS scale | 1.907 | 0.418 to 3.397 | 0.013 |
|  | EDMUS scale * Antibody diagnosis | -2.850 | -5.961 to 0.261 | 0.072 |
|  | Pain interference score | 2.430 | 1.328 to 3.533 | <0.001 |
|  | Pain interference score * Antibody diagnosis | 2.325 | 0.173 to 4.478 | 0.034 |
|  | HADS-A | 0.779 | 0.036 to 1.522 | 0.040 |
|  | HADS-A * Antibody diagnosis | 0.657 | -0.738 to 2.051 | 0.353 |
|  | HADS-D | 1.641 | 0.864 to 2.418 | <0.001 |
|  | HADS-D * Antibody diagnosis | -0.704 | -2.197 to 0.789 | 0.352 |
|  | Antibody diagnosis | 14.919 | -1.786 to 31.623 | 0.080 |

Antibody diagnosis: AQP4-Ab = 0, MOG-Ab = 1

Ab = antibody; AQP4 = aquaporin-4; EDMUS = European Database for Multiple Sclerosis; HADS-A = Hospital Anxiety and Depression Scale-Anxiety; HADS-D = Hospital Anxiety and Depression Scale-Depression; MFIS = Modified Fatigue Impact Scale; MOG = myelin oligodendrocyte glycoprotein.

**Table S3. Multivariable linear regression analysis (MFIS physical subscale) within AQP4-Ab and MOG-Ab patients separately, and as a combined cohort**

|  | Independent variable | Regression coefficient, *B* | 95% CI | p value |
| --- | --- | --- | --- | --- |
| AQP4-Ab | Age at MFIS assessment | 0.144 | 0.051 to 0.238 | 0.003 |
|  | Number of clinical attack/s | 0.475 | 0.047 to 0.903 | 0.030 |
|  | EDMUS scale | 1.084 | 0.362 to 1.806 | 0.004 |
|  | Pain interference score | 1.439 | 0.933 to 1.946 | <0.001 |
|  | HADS-D | 0.872 | 0.539 to 1.205 | <0.001 |
|  |  |  |  |  |
| MOG-Ab | Pain interference score | 1.322 | 0.397 to 2.247 | 0.006 |
|  | HADS-D | 1.144 | 0.620 to 1.668 | <0.001 |
|  | Fatigue-inducing comorbidities | 9.663 | 2.546 to 16.780 | 0.009 |
|  |  |  |  |  |
| Whole cohort | Age at MFIS assessment | 0.085 | 0.009 to 0.161 | 0.028 |
|  | Number of clinical attack/s | 0.378 | -0.0135 to 0.769 | 0.058 |
|  | EDMUS scale | 1.040 | 0.394 to 1.685 | 0.002 |
|  | Pain interference score | 1.461 | 0.988 to 1.934 | <0.001 |
|  | HADS-D | 0.908 | 0.609 to 1.207 | <0.001 |
|  | Fatigue-inducing comorbidities | 0.873 | -1.846 to 3.591 | 0.526 |
|  | Antibody diagnosis | 1.630 | -0.931 to 4.190 | 0.210 |

Antibody diagnosis: AQP4-Ab = 0, MOG-Ab = 1

Ab = antibody; AQP4 = aquaporin-4; EDMUS = European Database for Multiple Sclerosis; HADS-D = Hospital Anxiety and Depression Scale-Depression; MFIS = Modified Fatigue Impact Scale; MOG = myelin oligodendrocyte glycoprotein.

**Table S4. Multivariable linear regression analysis (MFIS cognitive subscale) within AQP4-Ab and MOG-Ab patients separately, and as a combined cohort**

|  | Independent variable | Regression coefficient, *B* | 95% CI | p value |
| --- | --- | --- | --- | --- |
| AQP4-Ab | Disease duration | -0.415 | -0.668 to -0.162 | 0.002 |
|  | Number of clinical attack/s | 0.858 | 0.265 to 1.451 | 0.005 |
|  | EDMUS scale | 1.110 | 0.434 to 1.786 | 0.002 |
|  | Pain interference score | 0.718 | 0.083 to 1.353 | 0.027 |
|  | HADS-A | 0.456 | 0.029 to 0.884 | 0.037 |
|  | HADS-D | 0.726 | 0.277 to 1.175 | 0.002 |
|  |  |  |  |  |
| MOG-Ab | Gender (Female) | -4.672 | -8.995 to -0.349 | 0.035 |
|  | Presence of severe attack/s | -6.013 | -10.911 to  -1.115 | 0.018 |
|  | HADS-A | 1.221 | 0.688 to 1.753 | <0.001 |
|  | Pain interference score | 3.159 | 1.755 to 4.563 | <0.001 |
|  | Pain severity score | -2.158 | -4.049 to -0.267 | 0.027 |
|  |  |  |  |  |
| Whole cohort | Disease duration | -0.267 | -0.461 to -0.073 | 0.008 |
|  | Number of clinical attack/s | 0.570 | 0.030 to 1.110 | 0.039 |
|  | EDMUS scale | 1.214 | 0.478 to 1.951 | 0.001 |
|  | Pain interference score | 1.207 | 0.540 to 1.873 | <0.001 |
|  | HADS-A | 0.529 | 0.172 to 0.886 | 0.004 |
|  | HADS-D | 0.716 | 0.325 to 1.108 | <0.001 |
|  | Gender (Female) | 0.275 | -2.715 to 3.264 | 0.856 |
|  | Presence of severe attack/s | -3.117 | -6.314 to 0.079 | 0.056 |
|  | Pain severity score | -0.532 | -1.192 to 0.129 | 0.114 |
|  | Antibody diagnosis | 0.379 | -2.665 to 3.423 | 0.806 |

Antibody diagnosis: AQP4-Ab = 0, MOG-Ab = 1

Ab = antibody; AQP4 = aquaporin-4; EDMUS = European Database for Multiple Sclerosis; HADS-A = Hospital Anxiety and Depression Scale-Anxiety; HADS-D = Hospital Anxiety and Depression Scale-Depression; MFIS = Modified Fatigue Impact Scale; MOG = myelin oligodendrocyte glycoprotein.
